# Hepatobiliary Cystadenocarcinoma

**DOI:** 10.1155/2012/298957

**Published:** 2012-11-01

**Authors:** Neal Wilkinson

**Affiliations:** Department of Surgical Oncology, Roswell Park Cancer Institute, Elm and Carlton Streets, Buffalo, NY 14263, USA

## Abstract

Biliary cystadenocarcinomas are rare tumors that are poorly understood. Preoperative imaging is imprecise and treatment is not standardized. We describe the presentation and treatment of one of these rare tumors and review the limited published literature.

## 1. Case Report

**Figure 1:**
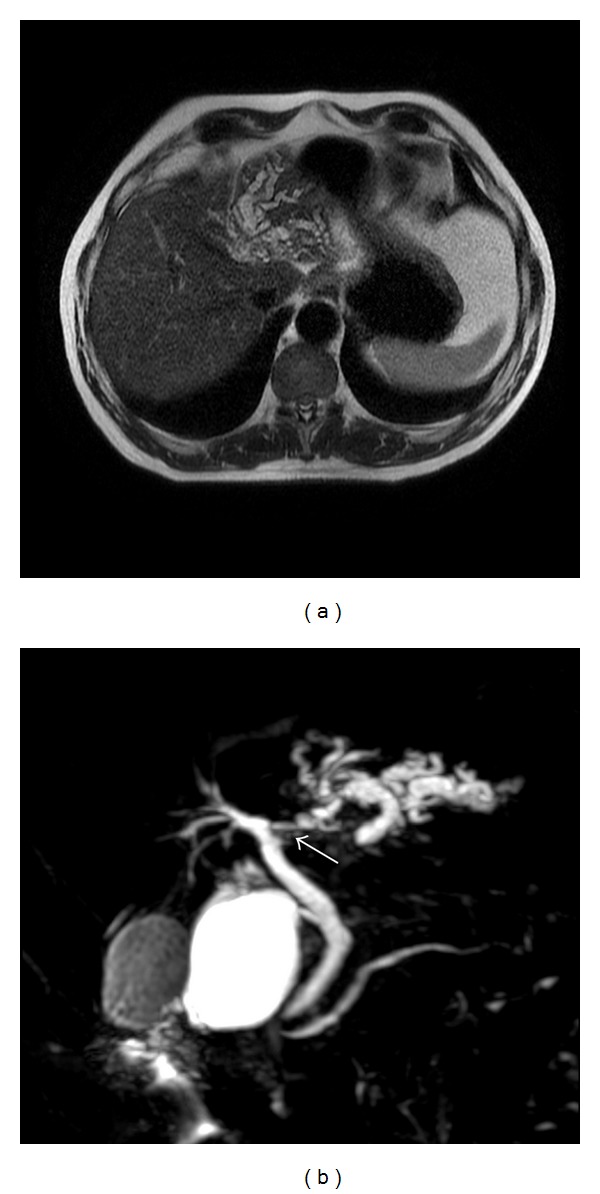
MRI demonstrates left hepatic biliary system is narrowed/occluded although a discrete mass is not well seen. MRCP demonstrates the cystic/dilated left biliary system with stricture/mass noted at the left and common bile duct junction (arrow).

**Figure 2:**
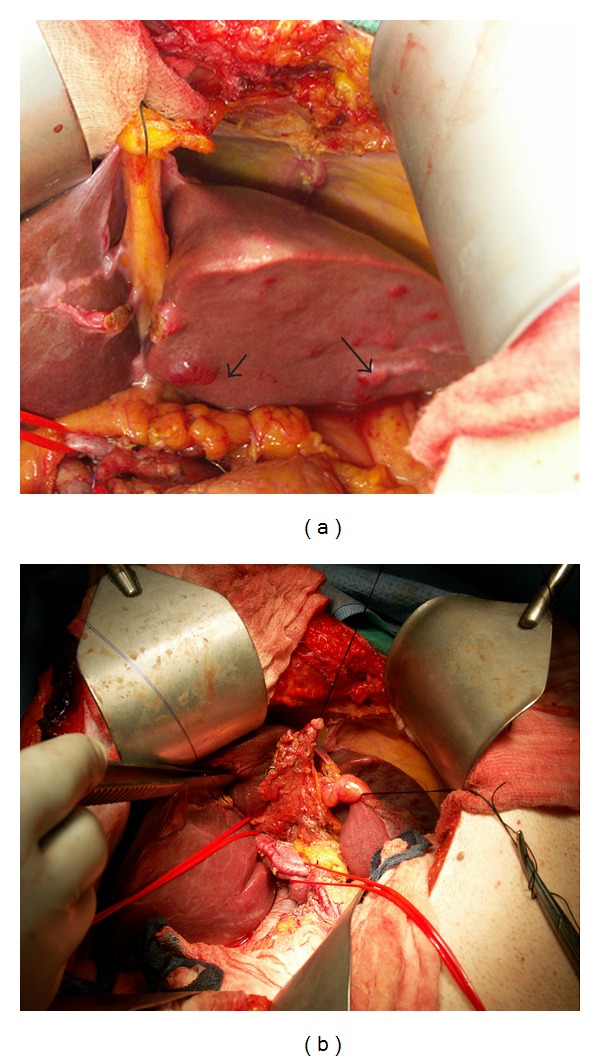
At exploration numerous cystic abnormalities were seen at the liver capsule (arrows). Initial impression was that these represented metastatic satellite lesion but proved to be simply ductal dilatation (a). Resection of the common bile duct with en bloc left hepatectomy, caudate resection, and regional lymphadenectomy was performed with a Roux en Y hepaticojejunostomy (b).

**Figure 3:**
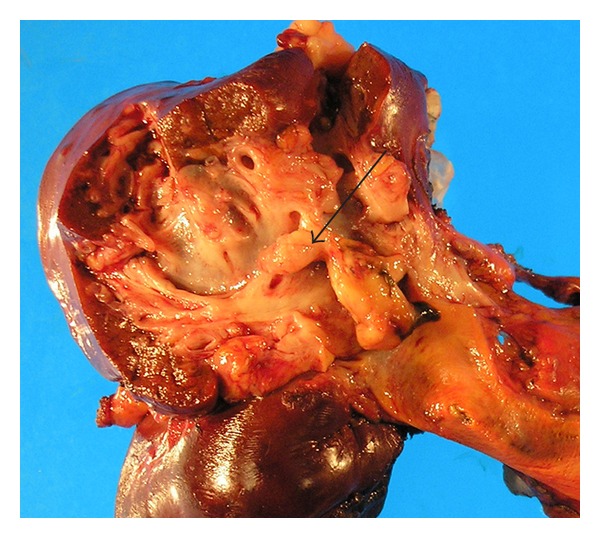
Resected specimen showing polypoid mass arising from a dilated biliary system (arrow).

**Figure 4:**
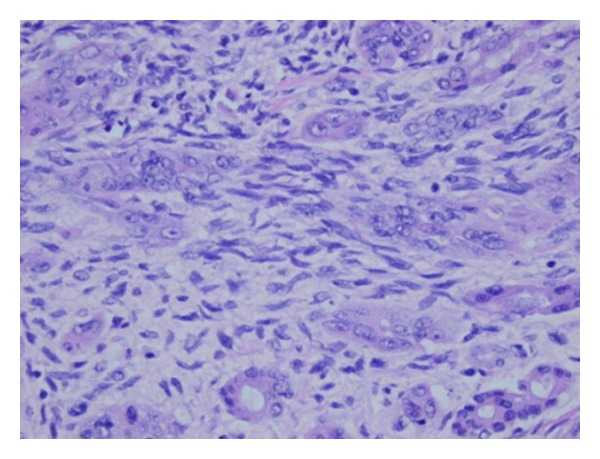
Histologic examination atypical glands (adenocarcinoma) infiltrating spindled mesenchymal stroma 200x.

 A 61-year-old male with no previous medical history, presented with right upper quadrant pain and mild elevation of total bilirubin to 2.9 and Ca 19-9 was elevated at 559 u/mL. All other laboratory values were within normal limits. Evaluation included a right upper quadrant ultrasound (U/S) and computed tomography (CT), which demonstrated left intrahepatic bile duct dilatation. No stones were noted in the biliary system or gallbladder. ERCP and MRCP demonstrated a mural nodule arising from confluence of the left hepatic duct and the common hepatic duct (Figure 1). Brushings were nondiagnostic. During staging laparotomy, what initially appeared to be metastatic satellite lesions proved to be dilated intra-hepatic biliary system (Figure 2, arrow head). The planned surgical resection was undertaken with curative intent: resection of the common bile duct with en bloc left hepatectomy, caudate resection, and regional lymphadenectomy. Reconstruction was performed with a Roux en Y hepaticojejunostomy to the secondary right hepatic bile ducts. Two exophytic polypoid tumor masses were identified within the left intrahepatic bile ducts, 2.5 and 2.9 centimeter in size (Figure 3). Microscopic analysis demonstrated well-differentiated adenocarcinoma arising within a hepatobiliary cystadenoma (Figure 4). Multifocal severe dysplasia was seen within the left intrahepatic biliary tree without evidence of invasive carcinoma. The remaining right biliary ducts and distal common bile duct were uninvolved. A 1 mm focus of metastatic disease was identified in the regional nodes (1/13). 

This patient was taken to surgery for treatment of a suspected intrahepatic cholangiocarcinoma not hepatobiliary cystadenocarcinoma. Radical resection with regional lymphadenectomy was performed with curative intent and frozen section only obtained at the right biliary margin (negative for dysplasia and cancer). Microscopic analysis demonstrated well-differentiated adenocarcinoma arising within a biliary cystadenoma with multifocal severe dysplasia. The malignant polyps contained atypical glands infiltrating into underlying mesenchymal (ovarian-like) stroma. A 1 mm focus of metastatic disease was identified with the lymphadenectomy specimen (1/13). The remainder of the resected biliary system, right biliary duct, and distal common bile duct were uninvolved. Adjuvant chemotherapy and radiation therapy were discussed but not recommended. Prognosis and outcome in some clinical series seem favorable in comparison to the highly fatal cholangiocarcinoma diagnosis. At two years following surgery, he remains disease-free with a normal computed tomography and Ca 19-9 level. 

## 2. Discussion

Biliary cystadenocarcinomas are tumors thought to arise from malignant transformation of biliary cystadenomas, but little is known about the risk or timing of malignant transformation. The biliary cystadenomas and cystadenocarcinomas were initially described by Edmondson [1] in 1958 and are classified as a primary liver tumor by the World Health Organization (WHO) [2]. Cystadenocarcinomas arise from the intrahepatic bile duct and are composed of multiloculated mucin producing epithelial cells. Malignant polypoid tumors and associated mesenchymal “ovarilan-like” stroma are classically seen. The tumors should be carefully distinguished from distinct entities such as primary bile duct cancer (cholangiocarcinoma) with dilated intrahepatic ducts and carcinomas arising from simple hepatic cyst [3]. There are also parallels and contrasts between these rare tumors and cystic neoplasms that arise in the pancreas: mucinous cystic tumors with ovarian stroma and intraductal papillary mucinous neoplasms (IPMN) [4]. Embryologic origins of pancreatic, hepatobiliary system, and ovaries have been drawn as a means to unify the similarities of these cystic tumors [5]. These reports provide fascinating descriptions but unfortunately little is conclusively known about these rare tumors.

These rare tumors are seldom identified accurately in the clinical evaluation of hepatic or biliary cysts. The criteria utilized to differentiate an atypical liver cyst from an adenoma/adenocarcinomas are nonspecific and include multiloculated cyst with internal septations, thickened or irregular cyst walls, mural and papillary projections, calcifications, or enhancement. Clearly not all atypical hepato-biliary cysts represent a cystic neoplasm. In one recent study, the combined sensitivity of CT, U/S, and FNA was 30% (true positives/true and false positives based upon imaging suspicion) [6]. Recent efforts have been made using modern computer tomography and contrast-enhanced ultrasonography to more accurately diagnose biliary cystic neoplasms [7, 8]. One of the largest surgical series of biliary cystadenomas was reported by Thomas et al. in 2005 [9]. They report on the diagnosis and outcome of 18 biliary cystadenomas and one cystadenocarcinomas treated over a 15-year period. The recommended management of suspicious liver cysts is complete surgical resection. Less invasive methods to include interval followup, drainage, or marsupialization are ineffective and may delay treatment of a malignant condition. In the series, the authors make no mention of suspicious lesions that were observed by nonhepatobiliary clinicians or of suspicious lesions that were resected that did not contain biliary cystadenomas (false positive). Some have estimated that up to 5% to 18% of asymptomatic patients have some form of liver cysts on imaging many with nonspecific high risk features. This stands in contrast to an estimated incidence of biliary cystadenoma at one in 20,000–100,000 and cystadenocarcinoma at one per 10 million. One could argue that the imbalance between the incidence of benign liver cysts and incidence of cystadenoma/carcinoma would make even the most sensitive radiographic test imprecise. At present, surgical resection should be recommended for all suspicious “biliary cystadenomas” but the clinician must understand the radiologic limitations. Hypothetically, the surgeons may have to remove 100 “suspicious” cysts to identify 30 precancerous biliary cystadenomas and treat one biliary cystadenocarcinoma.
